# Circular Health: exploiting the SDG roadmap to fight AMR

**DOI:** 10.3389/fcimb.2023.1185673

**Published:** 2023-06-22

**Authors:** Luca Mantegazza, Alessandra Mistral De Pascali, Olga Munoz, Costanza Manes, Alessandra Scagliarini, Ilaria Capua

**Affiliations:** ^1^ One Health Center of Excellence, University of Florida, Gainesville, FL, United States; ^2^ Section of Microbiology, Department of Medical and Surgical Sciences, Alma Mater Studiorum University of Bologna, Bologna, Italy; ^3^ Department of Wildlife Ecology and Conservation, University of Florida, Gainesville, FL, United States; ^4^ Johns Hopkins University, SAIS Europe, Bologna, Italy; ^5^ Institute of Food and Agricultural Sciences, University of Florida, Gainesville, FL, United States

**Keywords:** antimicrobial resistance (AMR), sustainable development goals (SDGs), sustainability, interdisciplinarity, one health (OH), circular health

## Abstract

Circular Health is a novel approach to address complex health issues that is based on the expansion of the One Health Paradigm. Circular health recognizes the need for a multidisciplinary convergence effort to complement the biomedical dimension of health. Antimicrobial resistance (AMR) is one of the greatest global concerns for public health that is likely on the rise, given the extensive use of antibiotics during the early Covid-19 years. Prior to the Covid-19 pandemic, an expert group chaired by Jim O’Neill published “The Review on Antimicrobial Resistance”, which contains a final report and recommendations on how to tackle AMR. The report, for the first time, considers AMR from a multi-perspective viewpoint highlighting how it cannot be successfully addressed unless there is a converging approach encompassing many dimensions of the problem. In this perspective, we propose to include the recommendations from that seminal report and other more recent reviews which include the lessons learnt from the Covid-19 pandemic, into the operational framework of the sustainable development goals (SDGs). AMR represents a perfect case study to explore how the SDG roadmap has the potential of becoming the driving force and implementation tool to address complex health issues by pursuing the optimization of resources and actions via a convergent and multi-stakeholder approach. The implementation of health-related policies through the whole spectrum of the SDGs could be both a novel and a well-established framework to inform multi-dimensional policies for more sustainable health in the future.

## Introduction

Antimicrobial resistance (AMR) is recognized as a leading cause of death around the world, one of the top ten global health priorities, and a threat to development (FAO et al., 2022). It requires urgent multisectoral action to achieve the Sustainable Development Goals (SDGs) (WHO, 2015). According to the United Nations Environment Programme (UNEP) it is imperative to tackle the environmental dimensions of AMR to maintain global progress towards the SDGs (United Nations Environment Programme, 2023). Thus, fighting AMR is essential not only to keep people and animals healthy, but also to achieve sustainability. In this paper we describe a new conceptual model, Circular Health, which seeks to address major health issues (such as AMR) by including relevant recommendations within the SDGs, thus achieving a convergence of efforts. For the reasons mentioned above, we used fighting AMR as a case study for our Circular Health model.

Our starting point was a seminal report that analyzed the magnitude and potential impact of AMR and the numerous steps necessary to address it published as the “Review on Antimicrobial Resistance” (O’Neill, 2016). However, after a fruitful beginning, global efforts sparked by that report have been completely sidelined by the efforts needed to manage the Covid-19 pandemic. For example, from March to October 2020, almost 80% of patients infected with Covid-19 in the USA received antibiotic treatment and estimates about increasing deaths caused by AMR are worrisome (CDC, 2022). A recent paper concludes that in many countries of the world the AMR issue is worsening (Tomczyk et al., 2021).

Hence, AMR is growing fast, and novel roadmaps should be developed to comprehensively address AMR by using existing consensus documents and frameworks to empower such activities. The 2030 Agenda for Sustainable Development is an existing framework that may be used to seek a broader convergence of AMR related efforts. Here we propose an example of how to implement a series of recommendations taken from the O’Neill report and selected others from more recent studies by including them in selected targets pertaining to each SDG, excluding, for obvious reasons, SDG 3 - Health and Well-being.

The aim of this paper is to exemplify how countries can contribute to fight AMR by implementing, expanding, or focusing on actions that are already part of the SDG roadmap.

## SDG1 no poverty

Poverty can be a lack of money, but also a lack of access (to resources, services, technology, and rights). Both types of poverty contribute to AMR. Poorer people are more susceptible to infectious diseases treated with antimicrobials (Do et al., 2021) and so is their livestock (McKune et al., 2021). Equally, as it is discussed in Aliro et al. (2021), lack of access to basic services such as health care and veterinary services promotes self-medication that greatly contributes to the development of AMR, especially when antimicrobials are cheap and available without prescriptions. For these reasons, reducing poverty would contribute to reducing AMR by itself. Moreover, access to health care and veterinary services should be explicitly listed as basic services in target 1.4, and the ability to live in healthy environments should be one of the dimensions of poverty of target 1.2.

## SDG2 zero hunger

The intensification of agriculture driven by increasing demand, especially for animal-source foods, is increasing antimicrobial use in livestock (Founou et al., 2021). The general balance of achieving sustainability while increasing production, already addressed in target 2.4, should include the role of antibiotics: they are important for maintaining the health and welfare of livestock and the economic stability of farms (Founou et al., 2021; Tandon and Bouzanis, 2021) but their misuse negatively affects human and animal health (Founou et al., 2021). Our recommendation is to explicitly include a rational use of antibiotics in food-producing animals as part of the meaning of “sustainable” in target 2.4 and develop specific monitoring indicators.

## SDG4 quality education

Lack of awareness and poor education are the main drivers of improper use and disposal of antimicrobials, in turn, one of the main causes of AMR. While certain categories of health care workers have inadequate or incorrect knowledge about how to appropriately manage antimicrobials and on the potential consequences of AMR (Ogunnigbo et al., 2022) most medical students show interest in learning more about “antimicrobial stewardship” after learning of the problem (Abbo et al., 2013). For this reason, we recommend extending the scope of target 4.7 to specifically include the importance of education on antimicrobial stewardship, AMR, and the appropriate and sustainable use of these drugs from primary through high school and in university programs in life sciences. The educational approach should be creative and interactive, especially for younger children, including the use of cartoons, posters and storytelling to influence AMR knowledge, beliefs and attitudes of young students and their parents (Appiah et al., 2022).

## SDG5 gender equality

Women represent 80% of caregivers worldwide (Sharma et al., 2016), thus playing a major role in the acquisition, administration, management, and disposal of drugs (including antibiotics and other antimicrobials); they are more susceptible to urinary tract infections and reproductive health issues treated with antimicrobials; and they are also the majority in those professions, such as teaching and health care, with more frequent exposure to infectious diseases (WHO, 2018). The World Health Organization (WHO) reports that AMR mitigation strategies with a gender focus are more people-centered and effective and thus it is reasonable to empower women to better understand AMR, especially for future generations, with a focus on how to contribute to its prevention (WHO, 2018). We propose to develop programs to empower women to understand and combat AMR in the framework of targets 5.4, 5.5, 5.6, 5.b, for example building upon existing initiatives such as HPV (human papillomavirus) prevention campaigns to increase awareness about AMR in collaboration with public health institutions, pharmacies, health care providers and drug manufacturers, using a combination of digital or conventional tools.

## SDG6 clean water and sanitation

Unsafe drinking water is one of the main sources of bacterial infections (Tandon and Bouzanis, 2021; Kaiser et al., 2022) and it is more common in low-resource settings, especially in low- and middle-income countries (LMICs) (Tandon and Bouzanis, 2021). When combined with self-medication and inappropriate disposal practices it is more likely to cause and amplify AMR. In addition, SDG6 includes equitable sanitation, hygiene for all and an end to open defecation, all essential to combat AMR. Promoting proper sewage systems also reduces the threat that water related to human uses, especially if not properly sanitized, poses for the environment, and the plants and animals found in it (Kaiser et al., 2022).

## SDG7 affordable and clean energy

Reliable access to electricity is fundamental to provide the highest level of care (Irwin et al., 2020). Indeed, electricity is required to run diagnostic services (O’Neill, 2016; Kaprou et al., 2021), store vaccines (Fanelli et al., 2022), which are instrumental to fighting AMR, and to administer alternatives to antimicrobial treatment such as hemofiltration devices (Kumar et al., 2021). Electricity is also necessary to efficiently run surveillance systems that require laboratory equipment and information technology (IT) infrastructure. Our recommendation is to expand target 7.1 to also measure the share of hospitals and health care facilities with access to reliable and affordable electricity. Moreover, target 7.b could be adjusted to prioritize investments in electrifying hospitals, diagnostic laboratories and health care centers.

## SDG8 decent work and economic growth

Wealthier countries, especially when experiencing rapid economic growth, tend to have a higher consumption of antimicrobials (Malik and Bhattacharyya, 2019). Since AMR has in turn a negative impact on economic growth (Dadgostar, 2019), it is important to expand target 8.1 to highlight the importance of developing best management practices for antimicrobials to achieve sustainable growth. Wealthier countries have the economic power to invert this tendency and pave the way for LMICs.

Health care is also one of the high value-added and labor-intensive sectors described in target 8.2. Investing in AMR mitigation strategies is necessary to increase the sector’s productivity and quality of services. At the same time, promoting better jobs and innovative enterprises in health care (target 8.5 and 8.3) is essential to address AMR. This positive feedback effect could be promoted by appropriately amending targets 8.2, 8.3, and 8.5. Moreover, managing the impact of AMR through the lens of economic policy could have beneficial effects given the number of proven tools already available to policymakers (Roope et al., 2019; Booth et al., 2020).

## SDG9 industry innovation and infrastructure

Scientific and technological innovation together with infrastructure development are the foundations of many policies to address AMR. The O’Neill report (O’Neill, 2016) highlights the importance of innovation to develop new antibiotics, vaccines, alternative treatments, rapid diagnostic tools, and to improve the global surveillance infrastructure. It also promotes the creation of a global innovation fund to support research on AMR. From an infrastructure point of view, target 9.4 should be expanded to include processing of antimicrobial waste (Booth et al., 2020; Kotwani et al., 2021). From a science and technology perspective, target 9.5 could be amended to include a focus on “anti-AMR” research and technology. Finally, target 9.3 could be amended to prioritize incubating start-up companies working on AMR, to generate the range of innovative ideas and technologies needed to address such a complex issue.

## SDG10 reduced inequalities

The global volume of antibiotic consumption (40.2 billion DDD -defined daily dose- in 2018) increased 46% since 2000 (Browne et al., 2021). However, this consumption was inequitable between high-income countries (HICs) and LMICs, with respective rates of 20.6 DDD and 13.1 DDD per 1000 population/day. The lowest rate was estimated for Sub-Saharan Africa despite high prevalence of sepsis.

Proper access to antibiotics for refugees and migrants is significantly influenced by the health systems of the host countries as reported by the WHO in 2022 (WHO, 2022a). Resistant bacteria often cause outbreaks in refugee camps, (Tokajian et al., 2018) posing a risk also for hosting communities. The concept of safe migration addressed by target 10.7, and specifically by the indicator 10.7.4, should be expanded to explicitly include proactive anti-AMR policies, especially in the context of refugee camps.

Promoting better public health practices to prevent infections, especially in poorer countries, will reduce antimicrobial consumption overall, including their misuse (Mendelson, 2022). Similarly, antimicrobial consumption can be reduced in wealthier countries by promoting greater equality of access and treatment among lower-income, undocumented, and/or uninsured people who are more likely to be exposed to infectious diseases and to self-medicate due to lack of access to health care providers (Nadimpalli et al., 2021). Reducing inter-country economic and health inequalities should be specifically included in target 10.b, while target 10.2 should be expanded to add inclusive access to health care and improved public health interventions.

## SDG11 sustainable cities and communities

A growing share of humanity, about 55% in 2020, are urbanite, highlighting the importance of building sustainable cities (United Nations Human Settlements Programme (UN-Habitat) 2020). Sustainability should include monitoring and promoting a healthy microbiome of the built environment (Bruno et al., 2022) because, on one hand, it exerts selective pressures on microorganisms to develop AMR, especially in health care settings (Tarricone et al., 2020; Bruno et al., 2022) but, on the other hand, it can also be a source of a healthy and balanced microbiome that helps city dwellers to fend off and/or control the growth of harmful microorganisms (Tarricone et al., 2020). The importance of a healthy microbiome could be included in target 11.7 as another important environmental factor of sustainable and healthy cities.

## SDG12 responsible consumption and production

Consumption behaviors and production practices of antimicrobials are among the main causes of AMR. From a consumption perspective, target 12.8 should be expanded to clearly promote increasing public awareness on how to properly use antimicrobials, how to dispose of them, and the risks associated with their misuse (Allison et al., 2017). This should go hand-in-hand with policies to control access and to reduce over-prescription. From a production perspective, target 12.4 should be expanded to include management of wastewater from factories producing antimicrobials or their ingredients: a major driver of AMR according to Kotwani et al. (2021).

## SDG13 climate action

There is a positive association between global warming and AMR (Kaba et al., 2020). Higher temperatures are associated with higher bacterial growth rates, increased gene transfer, and emergence of new pathogens (Kaba et al., 2020). Livestock farming, rising temperatures, and water and environmental contaminants were identified as factors linking AMR and climate change (Magnano San Lio et al., 2023). Some countries (e.g., Ireland) have divested from fossil fuels and invested into the Global Innovation Fund for AMR research, to tackle the climate crisis and AMR at the same time (Turner, 2018). Here we encourage the recognition of such a link on a global scale, so that the fight against both threats can be synergistic. Guidelines on AMR mitigation linked to livestock farming and environmental contamination should be updated and integrated in target 13.2 to encompass both climate change and AMR actions within national policies, strategies, and planning, providing solutions to reach a common goal.

## SDG14 life below water

Many human activities are drivers of AMR in marine life. Medicated feeds, a common practice in aquaculture, are an important source of AMR (Shah et al., 2014), and ocean plastics can accumulate and serve as vehicles for resistant bacteria, exacerbating their impact and spread (Moore and Millar, 2020). Contaminant discharge, particularly improper antibiotic disposal, contributes to increasing the load and density of resistant pathogens in coastal ecosystems, and consequent accumulation in marine species that can harbor resistant pathogens (Wallace et al., 2013). These drivers of AMR should be explicitly incorporated in targets 14.1, 14.2, and 14.6. Actions may include targeted screening of antimicrobials in municipal and agricultural wastewater, aquaculture, and marine debris to monitor misuse, estimate its threat, and tailor appropriate ocean AMR mitigation measures.

## SDG15 life on land

Protection of natural ecosystems can be significant in addressing AMR. On one hand, natural habitats can accumulate drugs discharged from hospitals, health care settings and agriculture, resulting in antimicrobial agents exerting selective pressure on environmental microorganisms (Radhouani et al., 2014). On the other hand, terrestrial wildlife can harbor and spread resistant pathogens (Greig et al., 2015) whose resistant genes can commonly and easily be exchanged between microbes affecting wildlife, humans, and domestic animals (Vittecoq et al., 2016). We invite to specifically include in the targets of SDG15 the benefit of reducing human encroachment on wild habitats on mitigating AMR drivers as well as developing monitoring plans for environmental disposal of antibiotics focused on local wildlife and the species-specific potential for antimicrobial accumulation and exchange.

## SDG16 peace, justice and strong institutions

Antibiotic misuse is one of the most important components of AMR and dysfunctional public institutions and corruption seem to account for some of the remarkable between-region variation in antibiotic consumption. Poor governance and corruption correlate better than antibiotic usage volumes with resistance rates (Collignon et al., 2015). A strong positive correlation between measures of corruption, in the health sector and in the society at large, has been shown in Europe (Rönnerstrand and Lapuente, 2017) as well as in several African countries (Harant, 2022). Furthermore, when the quality of governance is low, antibiotic stewardship programs are less effective, as well as food and water safety controls. Achieving targets 16.5 and 16.6 can contribute to controlling AMR. Moreover, increasing transparency can enable stakeholders, such as civil society, to demand accountability for results and be empowered as individuals to fight AMR.

## SDG17 partnership for the goals

AMR is a highly complex problem that in a globalized world is not bound to specific geographic areas. Therefore, fighting AMR requires an interdisciplinary and multi-stakeholder response in which every country participates (de Kraker et al., 2016). This means that wealthier countries have an interest in supporting economically and technologically those countries where a higher burden of infectious diseases and less awareness of AMR are paired with weaker governance and health systems (Gandra et al., 2020). This should be strengthened by creating South-South partnerships in collaboration with HICs partners. These collaborations need to address multiple issues such as inadequate laboratory infrastructure (Opintan et al., 2015) and surveillance systems (Hay et al., 2018), lack of comprehensive population-based surveillance (Gandra et al., 2020), and access to technology for data generation, analysis, sharing and dissemination (Pokharel et al., 2019). SDG17 should be expanded accordingly to increase international investments to support research and development of vaccines and newer drugs, as well as the transfer of technology and resources to promote: i) development and uptake of diagnostic tests; ii) improved surveillance systems; iii) computerized data repositories to collect and share surveillance data; iv) global efforts to improve antimicrobial stewardship through science- and evidence-based knowledge.

## Conclusion

**Figure 1:**
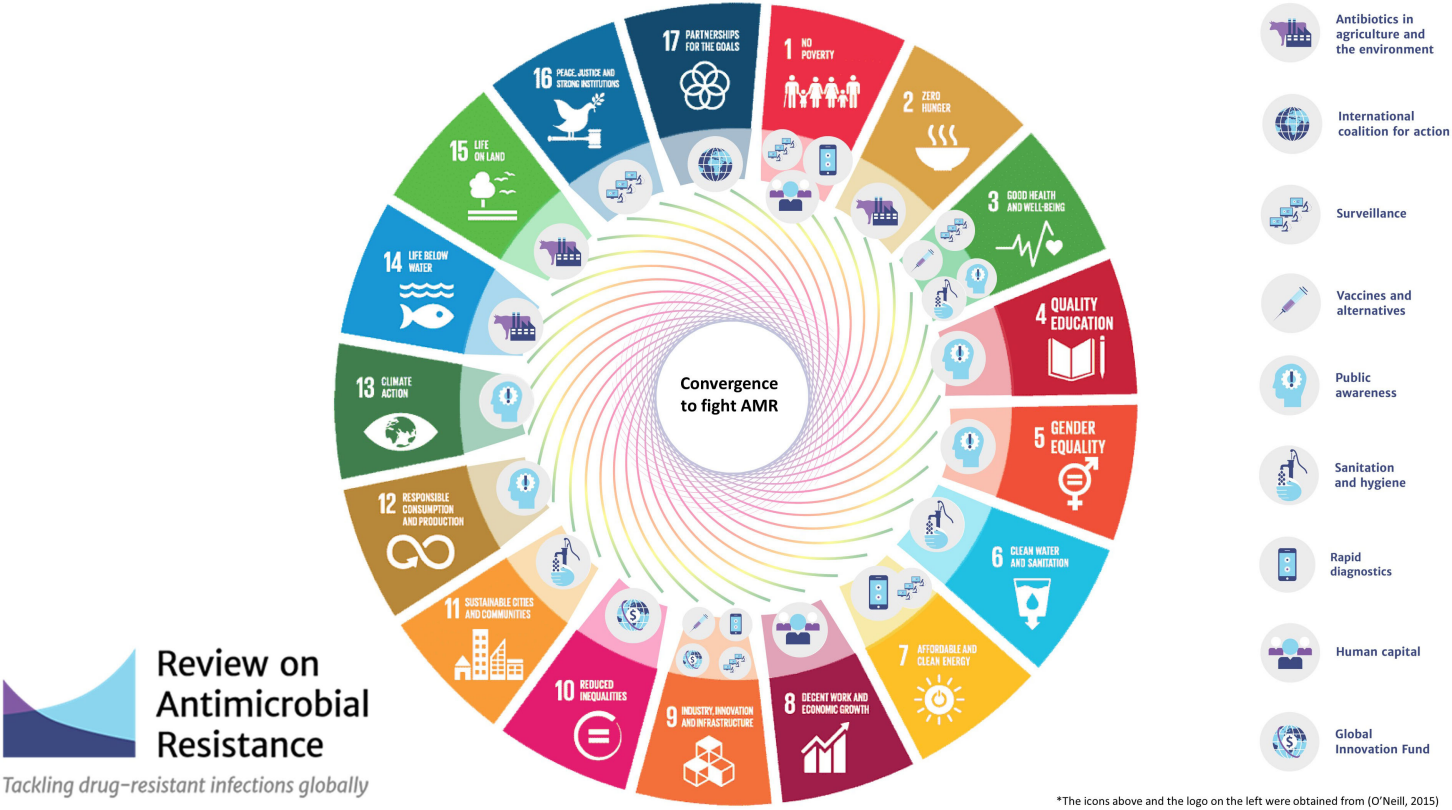
Mapping on the SDGs framework of the recommendations to fight AMR presented in O'Neill, 2016. Tackling drug-resistant infections globally: Final report and recommendations.

In this paper we propose a novel approach based on a convergence model to fight AMR, which exploits the existing SDG roadmap. Such a model is based on a circular approach to the problem that seeks to highlight win-win scenarios by enforcing existing objectives and targets to fortify the effort to combat AMR in a multidisciplinary synergistic effort (**Table 1**; **Figure 1**). If AMR is truly considered to be a global health priority and a hurdle to achieving sustainability, then it should also become a priority within the SDG roadmap. Efficiently fighting AMR with a Circular Health approach requires addressing this issue not only through the actions pertaining to SDG 3 - Health and Well-being, but also through the objectives of every SDG. Our examples can be a starting point to address AMR as a top health priority by funneling convergent and synergistic activities that are already part of the SDG roadmap or that can be included in a revised version of the roadmap. Such an effort could significantly advance health as a system by focusing on a major priority such as AMR.

**Table 1:** Recommendations to be included or implemented to adjust specific targets by SDG.

SDG	Target	Recommendation	References to supporting material
SDG1	Target 1.4: Access to basic services for all	Specify that basic services include affordable and easy access to health diagnostic services.	Impact on AMR of lack of access to basic services (Aliro et al., 2021)
SDG2	Target 2.4: “ensure sustainable food production systems [ … ]”	Explicitly include a rational use of antibiotics in food-producing animals as part of the meaning of “sustainable” and carefully monitor this.	Role of antibiotics in livestock production and impact on AMR (Founou et al., 2021)
SDG4	Target 4.7: Knowledge and skills for sustainable development	Education aimed at sustainability could be extended to include messages on AMR during both primary and higher education.	Awareness-raising interventions on students and their impact on AMR knowledge and beliefs (Appiah et al., 2022)
SDG5	Target 5.4: Unpaid care and domestic work Target 5.5: Women in leadership Target 5.6: Access to sexual and reproductive health Target 5.b: Enabling technology to empower women	Develop gender-tailored guidelines to increase awareness on appropriate use of antimicrobials, how to comply to treatment indications, and how to properly dispose of unused or expired antimicrobials.	Women are prescribed more antibiotics in primary health care than men (Schröder et al., 2016) Women represent over 70% of the health care worforce (Boniol et al., 2019) Women are more likely to be exposed to infections (reproductive health as well as professions dominated by women) (WHO, 2022b)
SDG6		Achieving SDG6 as it is will already contribute to reducing AMR.	Unclean water as an important source of infectious pathogens (Tandon and Bouzanis, 2021) Water “recognized as a primary environmental vector for AMR spread” (Kaiser et al., 2022). Limited focus of national action plans on the impact of AMR on the environmental sector, including bodies of water (Kaiser et al., 2022)
SDG7	7.1: Access to affordable and reliable electricity 7.b: “[ … ] Expand infrastructure and upgrade technology for supplying electricity for all in developing countries [ … ]”	Track proportion of hospitals and health care facilities with access to electricity (on top of proportion of general population). Prioritize electrification of hospitals and other health care facilities in developing countries.	Importance of electricity for: Best care (Irwin et al., 2020), diagnostic tools (O’Neill, 2016; Kaprou et al., 2021), vaccines (Fanelli et al., 2022), alternative treatments (Kumar et al., 2021)
SDG8	Target 8.2: Diversification and technology for higher levels of economic productivity Target 8.3: Policies to support decent job creation, entrepreneurship, innovation	Expand target 8.2 to address the importance of combating AMR to increase the productivity of the health care sector. Amend target 8.3 to promote the development of innovative companies in AMR related fields and the inclusion of AMR reducing policies in the “economic-oriented policies”.	Economic growth and antimicrobial (AM) consumption (Malik and Bhattacharyya, 2019) AMR and economic growth (Dadgostar, 2019)
SDG9	Target 9.3: Support for small-scale enterprises Target 9.4: Sustainable infrastructure and industries Target 9.5: Scientific research and technology in industry	Promote the incubation of start-ups developing “anti-AMR” technology. Include proper treatment of AM waste when retrofitting hospitals, pharmaceutical factories, and farms. Add a focus on AMR scientific research, especially new AM.	Importance of innovation to address AMR (O’Neill, 2016) Processing of AM waste (Booth et al., 2020; Kotwani et al., 2021)
SDG10	Target 10.2: Social, economic and political inclusion Target 10.7: Orderly, safe, regular and responsible migrations Target 10.b: Encourage development assistance and investment in least developed countries	Add inclusive access to health care and improved public health interventions for infection prevention to target 10.2. Explicitly apply the idea of “safe” migration to refugees, especially in the context of refugee camps and mass displacements (Indicator 10.7.4). Adjust target 10.b to explicitly aim to reduce inter-country inequalities in AMR education, awareness and surveillance capacity.	Link between access to health care services and AMR (Nadimpalli et al., 2021) Migrants and refugee camps: AMR (Tokajian et al., 2018), access to essential antibiotics for refugees and migrants (WHO, 2022a) Inequitable consumption of AM across countries (Browne et al., 2021) Public health interventions and reduction in AM use (Mendelson, 2022)
SDG11	Target 11.7: Access to safe, inclusive and accessible, green and public spaces for all	Monitor and ensure a healthy microbiome of the built environment. Integrate indicators related to the microbiome of the built environment.	AMR in hospitals (Bruno et al., 2022) Importance of microbial diversity and the ecosystem services that this could provide (Bruno et al., 2022)
SDG12	Target 12.4: Management of chemicals and all wastes Target 12.8: Spread relevant information and awareness for sustainable development	Expand target 12.4 to include management of wastewater from AM factories. Target 12.8 should include education on AMR to raise awareness about it.	Public awareness and AMR (Allison et al., 2017) Responsible production of AM (Kotwani et al., 2021)
SDG13	Target 13.2: “Integrate climate change measures into national policies, strategies and planning”	Guidelines should be updated and integrated to provide shared targets and solutions to these two global emergencies (climate change and AMR).	Resistant bacteria and AMR transmission at warmer temperatures (Kaba et al., 2020) The climate change - AMR link (Magnano San Lio et al., 2023) Report on a country divesting from fossil fuels (Turner, 2018)
SDG14	Target 14.1: Marine pollution Target 14.2: “By 2020, sustainably manage and protect marine and coastal ecosystems [ … ]” Target 14.6: Management of fisheries	Targets should be modified to explicitly address the main drivers of AMR in marine ecosystem settings: ocean plastics (14.1), improper antibiotic disposal (14.2), medicated aquaculture feeds (14.6). Example actions include targeted screening of AMs in wastewater, aquaculture, and marine debris to monitor misuse, estimate its threat, and tailor appropriate AMR mitigation measures.	Assessment of marine plastics as routes of AMR (Moore and Millar, 2020) Link between medicated feed in salmon aquaculture and AMR (Shah et al., 2014) Anthropogenic contaminant discharge and AMR (Wallace et al., 2013)
SDG15	Target 15.5: “Take urgent and significant action to reduce the degradation of natural habitats, halt the loss of biodiversity [ … ]”	Specifically include the development of a monitoring plan for environmental disposal of antibiotics at the water and soil level, with targeted research on the local wildlife and the species-specific potential for AM accumulation and exchange.	Terrestrial wildlife role in AMR transmission (Greig et al., 2015) Potential of wildlife as a reservoir of AMR (Radhouani et al., 2014) AMR exchange between wildlife, humans, and domestic animals (Vittecoq et al., 2016)
SDG16	Target 16.5: Substantially reduce corruption Target 16.6: Develop effective, accountable and transparent institutions	Clearly include addressing corruption and improving governance in the health care system. Highlight the importance of accountable and transparent institutions in addressing AMR.	Poor governance and AMR (Collignon et al., 2015) Corruption and AMR in Europe (Rönnerstrand and Lapuente, 2017) and Africa (Harant, 2022)
SDG17	Target 17.3 Mobilize financial resources for developing countries. Target 17.6: Knowledge sharing and cooperation for access to science, technology and innovation Target 17.7: “Promote the development, transfer, dissemination and diffusion of environmentally sound technologies to developing countries [ … ]”	Expand target 17.3 to clearly state the importance of increasing international investments in research and development of vaccines and newer drugs. Highlight in target 17.6 the importance of international cooperation and development of international IT and data systems to combat AMR. Expand target 17.7 to include necessary technology to combat AMR.	Tackling AMR in LMICs (Pokharel et al., 2019) Challenges of AMR surveillance in LMICs (Gandra et al., 2020)

## Data availability statement

The original contributions presented in the study are included in the article/supplementary material. Further inquiries can be directed to the corresponding author.

## Author contributions

IC conceived the contents and outline of the manuscript. All authors were assigned specific sections of the manuscript and identified the literature relevant for their sections. LM wrote the first draft of the manuscript assembling and synthetizing the materials provided by all authors. AP curated the bibliography. All authors contributed to the article and approved the submitted version.
